# Purification and Structural Characterization of Polysaccharides from *Polygonum multiflorum* Thunb. and Their Immunostimulatory Activity in RAW264.7 Cells

**DOI:** 10.3390/foods13060932

**Published:** 2024-03-19

**Authors:** Yan Gou, Donglin Gu, Jing Fan, Jianbo Yang, Ke Zan, Jingjing Liu, Hongyu Jin, Ying Wang, Feng Wei, Shuangcheng Ma

**Affiliations:** 1 NMPA Key Laboratory of Quality Evaluation of Chinese Patent Medicines, Sichuan Institute for Drug Control, Chengdu 611731, China; gouyan@scsyjs.org (Y.G.); 13348821019@163.com (D.G.); 2 Institute for Control of Chinese Traditional Medicine and Ethnic Medicine, National Institutes for Food and Drug Control, Beijing 102629, China; fanjing092021@163.com (J.F.); yangjianbo82@nifdc.org.cn (J.Y.); zanke@nifdc.org.cn (K.Z.); 18911002859@126.com (J.L.); jhyw@nifdc.org.cn (H.J.); weifeng@nifdc.org.cn (F.W.); 3 Chinese Pharmacopoeia Committee, Beijing 100061, China

**Keywords:** *Polygonum multiflorum* Thunb., purification, polysaccharides, structural characterization, immunostimulatory

## Abstract

*Polygonum multiflorum* Thunb. (PM) and derived products are broadly utilized in Chinese traditional medicine. According to our previous research, PM mostly contains polysaccharides, which display a wide range of biological activities. Two water-soluble polysaccharides (PMPs-1 and PMPs-2) were obtained from PM by DEAE-Cellulose and Sephadex G-100 column chromatography. Colorimetry, HPGPC-MALLS-RID, HPLC-PDA, methylation, FT-IR, NMR, and SEM were used to characterize these polysaccharides. PMPs-1 and PMPs-2 had average molecular weights of 255.5 and 55.7 kDa, respectively. PMPs-1 consisted of Man, Glc, Gal, and Ara at 0.9:78.6:1.0:1.6 and was a glucan with → 4)-Glc*p*-(1 → as a backbone. Meanwhile, PMPs-2, an acidic polysaccharide, comprised Rha, GalA, Glc, Gal, and Ara at 3.2:20.3:2.7:1.0:8.3. PMPs-1 and PMPs-2 significantly improved the proliferation of RAW 264.7 cells and induced NO, TNF-α, and IL-6 release. This study reveals that these two polysaccharides can be explored as novel immunomodulators and provide a basis for further development of PM in food and pharmaceutical industries.

## 1. Introduction

The food market has experienced a profound and rapid transformation due to increasing interest among individuals in natural products, which harbor demonstrated health-enhancing properties. Polysaccharides, which are abundant in living organisms, represent a prevalent category of large molecular compounds with a long history of application within the food industry. Multiple reports have demonstrated diverse biological activities for plant polysaccharides, including immune regulation, antioxidant effects, hypoglycemic properties, anti-tumor activity, hypolipidemic activity, and anticoagulant effects, as well as relatively low toxicity [1,2,3,4]. These benefits indicate the significant potential of plant polysaccharides to be used in the development of healthy foods and medicinal preparations [5]. With the market demand as a driving force, researchers are actively exploring novel polysaccharides derived from animals, plants, and microorganisms for therapeutic benefits [6,7].

*Polygonum multiflorum* Thunb. (PM) root, commonly referred to as “Heshouwu” in China, has a widespread distribution across the globe. With a rich history spanning hundreds of years, PM and derived products are considered prominent herbal preparations in China and numerous East Asian countries [8]. A comprehensive analysis of the available evidence indicates that PM can be used to alleviate aging, hyperlipidemia, cancer, and inflammation, showing great potential in the field of drug and health care product development [9]. Moreover, this botanical remedy exhibits additional benefits, including immune regulatory capabilities, neuroprotective effects, and the ability to alleviate various other ailments. Its multifaceted nature positions PM as a promising candidate for addressing complex health conditions. The fundamental constituents that contribute to the efficacy of PM primarily comprise stilbene glycosides, anthraquinones, phenols, phospholipids, and polysaccharides [10,11,12]. Polysaccharides make up more than 2% of PM, representing its main components. Polysaccharides regulate immunity and antioxidant activity. In a study promoted by Zhang et al., two polysaccharides, namely, WPMP-1 and WPMP-2, were purified from raw PM. Composition analysis revealed that WPMP-1 consisted exclusively of glucan (100%), while WPMP-2 exhibited a complex composition comprising arabinose (48%), rhamnose (23%), galactose (15%), and galacturonic acid (14%). To examine the potential immunomodulatory effects of these polysaccharides, researchers attempted to evaluate their effects on the proliferation of splenocytes and the phagocytic properties of peritoneal macrophages. These investigations aimed to determine whether WPMP-1 and WPMP-2 effectively activate these vital immune functions providing compelling evidence indicating the immunomodulatory potential of polysaccharides derived from PM. PM polysaccharides predominantly comprise neutral sugars, namely, glucan or arabinose, constituting a substantial majority of over 60% in total composition [13]. However, there is no systematic structural analysis and immunological activity study on the acidic polysaccharides from PM.

In this study, two water-soluble polysaccharides extracted from PM were characterized, including PMPs-1 and PMPs-2. The characterization process involved a comprehensive array of analytical techniques, such as HPLC, high-performance gel permeation chromatography/multi-angle laser light scattering/refractive index detector (HPGPC-MALLS-RID), methylation analysis, scanning electron microscopy (SEM), Fourier-transform infrared spectroscopy (FT-IR), and nuclear magnetic resonance (NMR). By systematically investigating the immunopotentiation abilities of these polysaccharides in RAW 264.7 cells, this study aimed to provide deeper insights into the intricate associations of the structural features of PMPs-1 and PMPs-2 with their pharmacological properties. The outcomes of this study provide valuable guidance for harnessing the potential of PM polysaccharides as functional food ingredients or immunomodulatory agents, promoting their enhanced utilization in various applications.

## 2. Materials and Methods

### 2.1. Materials

The PM was collected at a local market in Henan province, China. DEAE cellulose-52 and Sephadex G-100 were provided by Solarbio (Beijing, China). The National Institute for Food and Drug Control (Beijing, China) provided rhamnose (Rha), glucuronic acid (GlcA), galactose (Gal), mannose (Man), glucose (Glc), and arabinose (Ara) standards. Trifluoroacetic acid (TFA) was distributed by Oka (Beijing, China). Sodium borodeuteride (NaBD_4_) and 1-phenyl-3-methyl-5-pyrazolone (PMP) were provided by Aladdin (Shanghai, China). The remaining reagents were of analytical grade. RAW 264.7 macrophages were from the BeNa Biotechnology Research Institute based in Beijing, China. The assay kits for NO, TNF-α, and IL-6 detection were provided by Jiangsu Enzyme-free Industrial, headquartered in Jiangsu (Nanjing, China).

### 2.2. Extraction, Isolation, and Purification of PM Polysaccharides

#### 2.2.1. Extraction of PRMPs

Polysaccharide extraction was based on water decoction and alcohol precipitation [14]. Briefly, samples were added to 80% ethanol and heated for 1 h at 85 °C. After drying, extraction was performed with water (1:20, *m*/*v*) for 2 h at 100 °C. The samples were centrifuged (5000 rpm for 10 min) and supernatants were concentrated by evaporation to 10 mL. Next, ethanol was added to a final concentration of 80% (*v*/*v*) for overnight incubation at 4 °C. The precipitates obtained by centrifugation underwent an ethanol wash. The Sevag’s method was utilized to remove proteins. Small molecular compounds were removed with molecular weight retention centrifuge tubes (10 k MWCO, Thermo, Beijing, China). Finally, the precipitates were lyophilized to obtain the polysaccharide of PM (RPMPs).

#### 2.2.2. Separation and Purification of RPMPs

DEAE cellulose-52 anion exchange column chromatography was employed (2.6 × 40 cm) to separate and purify RPMPs. Elution utilized ultrapure water containing 0.3 mol/L NaCl as the eluent, at 3 mL/min, ensuring efficient separation of the desired components. To monitor the purification progress, each fraction was collected during elution, with a collection volume of 10 mL/tube. Finally, the obtained fractions were analyzed by the phenol-sulfuric acid method to detect polysaccharides. 

After the initial separation, the fractions containing the target polysaccharides were concentrated by employing a rotary evaporator under reduced pressure, at 50 °C. To further refine the purification, the concentrated samples were using a Sephadex G-100 column with dimensions of 2.6 × 40 cm. This step utilized ultrapure water as the eluent, at 0.5 mL/min, aiming to separate different components based on their molecular sizes and properties. Throughout the fractionation process, 10 mL fractions were obtained and preserved for subsequent analysis. The same analytical methods mentioned above were employed to analyze the collected fractions. After concentration, the samples were dialyzed with ultrapure water at 4 °C for 48 h, and PMPs-1 and PMPs-2 were obtained by vacuum freeze-drying.

### 2.3. Structural Characterization of Polysaccharides

#### 2.3.1. Analytical Methods

Quantification of total sugar content was carried out by the phenol-sulfuric acid method, and glucose was employed as a reference standard for calibration [15]. Furthermore, uronic acid quantitation utilized the M-hydroxybiphenyl method, with galacturonic acid (GalA) as a reference standard for calibration [16]. 

#### 2.3.2. *M*w Assessment

To determine the *M*w of PMPs-1 and PMPs-2, HPGPC-MALLS-RID was carried out, as outlined previously [17]. During the analysis, elution was carried out by employing a mobile phase consisting of 0.1 mol/L NaCl at 0.5 mL/min.

#### 2.3.3. Monosaccharide Compositions

Derivatization of samples using PMP (1-phenyl-3-methyl-5-pyrazolone) was based on an established method [18]. In this process, PMPs-1 and PMPs-2, at a concentration of 1 mg/mL, were subjected to hydrolysis using TFA at a concentration of 4 mol/L. Hydrolysis was performed in sealed tubes under nitrogen at 120 °C for 2 h. TFA was removed by washing with methanol three times, and the hydrolysate was dissolved in ultrapure water (1 mL). Subsequently, a reaction mixture was prepared with 1 mL of the polysaccharide hydrolysate, 300 μL of a methanol solution containing the PMP at 0.5 mol/L, and 150 μL of 0.25 mol/L NaOH. The reaction was carried out under controlled conditions at 70 °C for 2 h. Finally, 150 μL of a 0.25 mol/L HCl solution was used to stop the reaction at ambient temperature. 

The mixture was extracted with 1 mL chloroform and repeated three times. The supernatant was filtered through a 0.45 μm pore membrane for high-performance liquid chromatography (HPLC). The HPLC system employed was manufactured by Waters (Acquity H-class, Waters, Milford, MA, USA). HPLC conditions were as follows: ZORBAX Eclipse XDB-C_18_ column (100 × 2.1 mm, particle size of 1.8 μm) provided by Agilent (Santa Clara, CA, USA); mobile phase, 84% solution A (0.1 mol/L KH_2_PO_4_ at a pH of 6.9) and 16% B (acetonitrile); detection wavelength, 250 nm; column temperature, 25 °C; flow rate, 0.3 mL/min; injection volume, 10 μL.

#### 2.3.4. Methylation Analysis

Methylation analysis was performed using the Hakomori method [19], with slight modifications. Firstly, PMPs-2 (10 mg) was reduced using NaBD_4_ and repeated three times. Notably, this process involved the introduction of deuterium-labeled methylene groups onto the carboxyl carbon of GalA. Consequently, GalA quantification was performed by examining the *m*/*z* of Gal via GC-MS. The reduction products of PMPs-1 and PMPs-2 underwent methylation. This involved three treatment cycles with 2 mL of CH_3_I at ambient temperature. Next, the samples were incubated with TFA (2 mol/L) at 120 °C for 2 h. The resulting hydrolysates were then subjected to reduction by NaBD_4_. Acetylation was carried out by incubation with pyridine and acetic anhydride. The partially methylated alditol acetates (PMAAs) were assessed with a GC–MS system (GCMS-TQ8050 NX, Shimadzu, Kyoto, Japan) equipped with a mass spectrometer and an SP-2330 capillary column (30 m × 0.25 mm, 2 μm), with a starting temperature of 80 °C, increased by 30 °C/min to 170 °C, and then by 4 °C/min to 240 °C, which was finally held for 10 min. The carrier gas He was used at 1.10 mL/min. The split ratio and inlet, ion source, and ion interface temperatures were 10 °C, 230 °C, 230 °C, and 230 °C, respectively. The injection volume was 1 μL. In mass spectrometry, the scan mode was used to collect data, with a scan ion range of *m*/*z* 50~800.

#### 2.3.5. Infrared Spectral Analysis

FT-IR spectra were acquired on a Nicolet Nagna-IR 550 spectrophotometer (Thermo Fisher Scientific, Waltham, MA, USA), spanning from 4000 to 400 cm^−1^. Firstly, PMPs-1 and PMPs-2 were ground alongside spectroscopic grade potassium bromide (KBr) powder. The resulting blend was pressed to form compacted pellets with a uniform thickness of 1 mm. These pellets served as the sample for FT-IR measurements, enabling comprehensive spectral characterization.

#### 2.3.6. SEM

PMPs-1 and PMPs-2 were analyzed with a QUANTA 250 FEI Scanning Electron Microscope (Hillsboro, OR, USA). A 5 mg polysaccharide sample was placed on a conductive carbon film with a double-sided adhesive and transferred to the ion sputtering instrument for a 40 s gold spraying. Finally, each sample was examined by SEM at 5 kV.

#### 2.3.7. NMR Analysis

NMR spectra for PMPs-1 and PMPs-2 were generated with a high-field 600 MHz NMR spectrometer (Bruker, Billerica, MA, USA). Briefly, 50 mg polysaccharide specimens were added to 0.5 mL deuterium oxide (D_2_O) for NMR analysis. The NMR study included recording both ^1^H and ^13^C spectra and was conducted at 30 °C. Subsequently, the acquired NMR data were subjected to comprehensive analysis using the MestReNova 6.1.0.

### 2.4. Immunoregulatory Activity Test

RAW 264.7 cells were procured from the BeNa Biotechnology Research Institute headquartered in Beijing, China. Dulbecco’s modified Eagle’s medium (DMEM) manufactured by Gibco (Billings, MT, USA) was utilized for cell culture after supplementation of 10% heat-inactivated fetal bovine serum (FBS). Cells were cultured with 5% CO_2_ at 37 °C.

#### 2.4.1. Cell Viability

To assess the potential cytotoxicity of PMPs-1 and PMPs-2 in RAW 264.7 cells, an MTT (3-(4,5-dimethyl-2-thiazolyl)-2,5-diphenyl-2H-tetrazolium bromide) kit (Abcam, Cambridge, UK) was employed. RAW 264.7 cells at 2.5 × 10^5^/mL were seeded in a 96-well plate. Subsequently, these cells were incubated at 37 °C in a humid environment with 5% CO_2_ for an appropriate duration. Following this preincubation phase, the culture medium was aspirated and replaced by fresh media with various concentrations (0 [blank], 25, 50, and 100 μg/mL) of both PMPs-1 and PMPs-2. Each concentration was assessed in six independent replicates. After incubation for 24 h, 20 μL of MTT (5 mg/mL) was added per well for a 4 h incubation at 37 °C. Following the incubation period, an absorbance reading at 450 nm was taken using a microplate reader. Subsequently, cell viability was assessed as follows: macrophage cell viability (%) = (A_1_/A_0_) × 100%, where A_0_ denotes the absorbance value obtained from the blank control group (serving as a reference baseline) and A_1_ pertains to the absorbance value acquired exclusively from the sample, reflecting the specific response or signal exhibited by the investigated substances.

#### 2.4.2. Determination of the Levels of NO and Cytokines

NO, TNF-α, and IL-6 amounts were quantified in RAW 264.7 cells with ELISA kits as directed by the respective manufacturers. Briefly, 100 μL of a RAW 264.7 cell suspension at 10^6^ cells/mL were seeded into individual wells of a high-quality 96-well plate. Subsequently, RAW 264.7 cells underwent a 24 h treatment with varying amounts (25, 50, 100 μg/mL) of PMPs-1 and PMPs-2. For comparison, a blank control consisting of DMEM and the positive control (LPS at 1 μg/mL) were also examined. After treatment, each cell culture supernatant was collected for further analysis. NO, TNF-α, and IL-6 levels were determined by ELISA, strictly following the instructions outlined in each ELISA kit’s manual. 

### 2.5. Statistical Analysis

Data are mean ± standard deviation (SD). One-way ANOVA was performed to compare groups with SPSS 20.0 based on triplicate assays. *p* < 0.05 reflected statistical significance.

## 3. Results and Discussion

### 3.1. Purification and Chemical Composition of Polysaccharides

**Figure 1:**
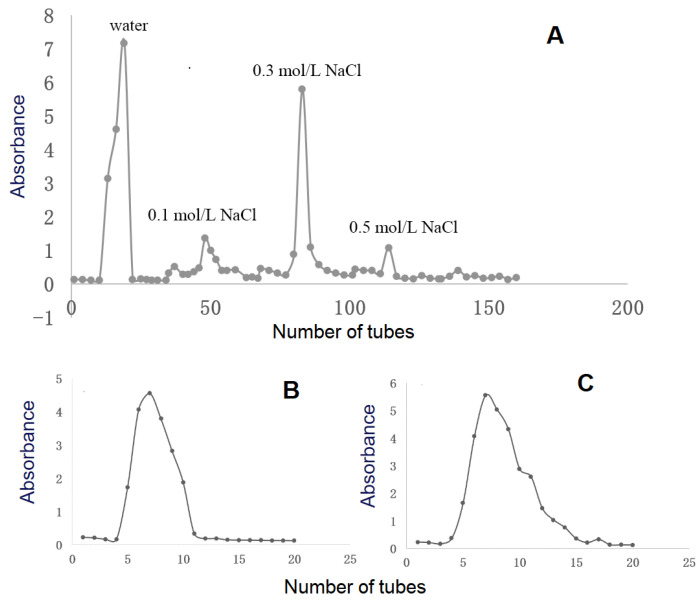
Isolation and purification of PMPs-1 and PMPs-2 from *Polygonum multiflorum* Thunb. The elution profile of RPMPs on a DEAE cellulose-52 chromatography column (**A**). The elution profiles of RPMP-WA (**B**) and RPMP-0.3A (**C**) on a Sephadex G-100 chromatography column.

Crude RPMPs were isolated by DEAE cellulose-52 anion exchange column chromatography as two major peaks, i.e., RPMP-WA and RPMP-0.3A (Figure 1A), which underwent further purification with a Sephadex G-100 column, respectively (Figure 1B,C). Finally, two heteropolysaccharides were obtained, namely, PMPs-1 and PMPs-2, making up 5.6% and 21.5% of the crude polysaccharide fractions of RPMPs, respectively.

**Figure 2:**
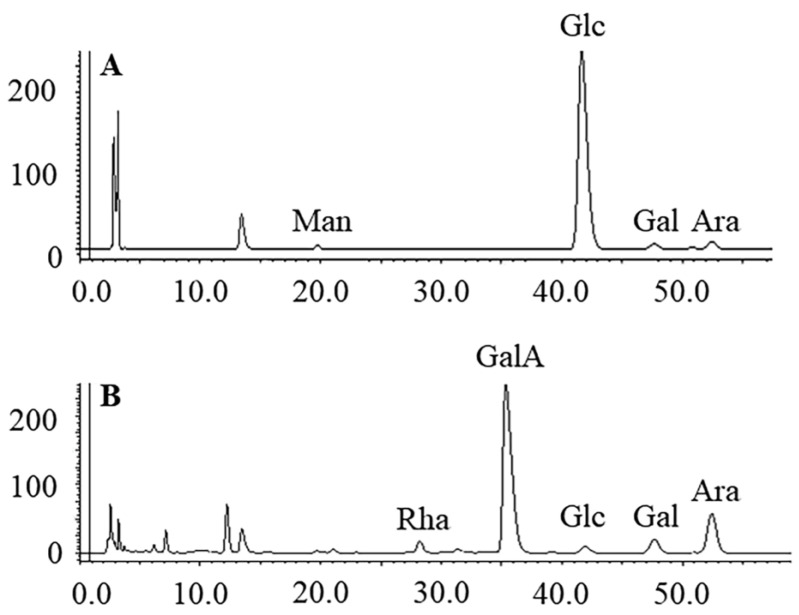
HPLC analysis of PMP derivatives of PMPs-1 (**A**) and PMPs-2 (**B**).

Utilizing the phenol-sulfuric acid method and employing glucose as a reference, PMPs-1 and PMPs-2 exhibited neutral sugar contents of 95.5 ± 3.55% and 19.7 ± 2.42%, respectively. Furthermore, the quantification of uronic acid content in PMPs-1 and PMPs-2 was accomplished by the M-hydroxybiphenyl method, using galacturonic acid as a reference. The results indicated that PMPs-1 had a uronic acid content of 4.7 ± 2.12%, which was substantially lower than that of PMPs-2 (79.4 ± 3.11%). Additionally, UV spectra revealed that neither polysaccharide exhibited absorption at 280 nm, thereby signifying the absence of proteins [20]. PMP-HPLC-PDA was performed to assess the monosaccharide compositions of both PMPs-1 and PMPs-2, and the results are depicted in Figure 2. Intriguingly, PMPs-1 and PMPs-2 exhibited distinct monosaccharide profiles. PMPs-1 was composed of Man (0.9), Glc (78.6), Gal (1.0), and Ara (1.6). On the other hand, PMPs-2 had an entirely different arrangement of monosaccharides, comprising Rha (3.2), GalA (20.3), Glc (2.7), Gal (1.0), and Ara (8.3) in their respective molar ratios. The complexity of these monosaccharides suggests that PMPs-1 and PMPs-2 differ in terms of underlying molecular structure.

### 3.2. Molecular Properties of PMPs-1 and PMPs-2

**Figure 3:**
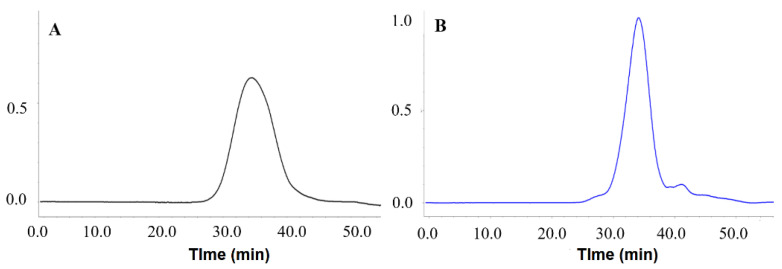
(**A**) *M*w analysis of PMPs-1 using HPGPC-MALLS-RID; (**B**) *M*w analysis of PMPs-2 using HPGPC-MALLS-RID.

The HPGPC chromatograms are shown in Figure 3, where both polysaccharides exhibited a symmetrical peak. However, the *M*w values of PMPs-1 and PMPs-2 were different. PMPs-1 showed an *M*w of 255.5 kDa, which was higher than the 55.7 kDa determined for PMPs-2. It is worth noting that the *M*w results of this study differ somewhat from previous findings. For instance, Lv et al. reported *M*w values of 480 and 610 kDa, respectively, for two purified polysaccharide components [21]. Meanwhile, *M*w values of 2.04 and 92.13 kDa were reported for two purified polysaccharides by Zhang et al., respectively [13]. The *M*w measurement results for PM polysaccharides were inconsistent, which may be related to divergent separation and *M*w determination methods. In the determination of *M*w of polysaccharides by using GPC-RID, well-characterized polysaccharide standards, such as commercially available pullulans or dextrans, should be used in the GPC column calibration. In studies by Lv et al. and Zhang et al., the GPC-RID method with polysaccharide standards was utilized to obtain relative *M*w measurements. On the other hand, the present study adopted the HPGPC-MALLS-RID approach, which is a method for determining absolute *M*w without relying on reference polysaccharide materials [22,23].

The evaluation of polysaccharide uniformity can be accomplished by analyzing their *PDI* (*M*w/*M*n) values, where a value close to 1 indicates a high degree of homogeneity. In the case of PMPs-1 and PMPs-2, both showed *PDI* values of approximately 1.21 and 1.14, respectively. These values indicate that the *M*w distribution of PMPs-1 and PMPs-2, along with their derivatives, exhibited a remarkable level of consistency and uniformity. The proximity of these *PDI* values to 1 reinforces the notion that the polysaccharides in question possess homogeneous *M*w values without significant variations or deviations. Such findings shed light on the intrinsic stability and regularity of the polysaccharide structures under investigation. 

### 3.3. Methylation Analysis of PMPs-1 and PMPs-2

The glycosidic linkages and branching of the polysaccharides were obtained by traditional methylation analysis. PMPs-1 displayed four peaks in the total ion chromatogram, indicating the presence of specific glycosidic linkages. On the other hand, PMPs-2 exhibited a more complex profile with eight distinct peaks, suggesting a broader range of glycosidic linkages within its structure compared with PMPs-1. Considering various analytical indexes, including the relative retention time and peak fragments (Supplementary File S1), and using the comprehensive Complex Carbohydrate Research Center (CCRC) Spectral Database for partially methylated alditol acetate (PMAA), the glycosidic linkages of PMPs-1 and PMPs-2 were determined. The results are summarized in Table 1, providing a comprehensive and systematic overview of the specific glycosidic linkages in each polysaccharide. 

Results showed that the linkage types in PMPs-1 were 1,4-linked-Glc*p*, 1,6-linked-Glc*p*, 1,4,6-linked-Glc*p*, and T-linked-Glc*p*, at ratios of 80.54:2.81:8.62:8.03. These results suggested that PMPs-1 had a backbone composed of → 4)-Glc*p*-(1 → units, which was consistent with previous findings [13]. Notably, the analysis of the polysaccharide structure suggested a ratio between terminal units and branching points of 1:1. 

There are more GalA units in PMPs-2; due to the poor solubility of acidic polysaccharides in methylation analysis, many hydroxyl groups could not participate in the methylation reaction, resulting in a reduced degree of methylation, and could not accurately determine the linkage sites of acidic sugars. Many hydroxyl groups could not participate in the methylation reaction, resulting in a reduced degree of methylation and the inability to accurately determine the linkage sites of acidic sugars. Therefore, GalA was esterified and reduced to Gal using the carbodiimide reagent, and the linkage sites of acidic sugars were inferred from the linkage sites analyzed by methylation after esterification and reduction. GC-MS analysis of PMPs-2 revealed at least nine easily identifiable methylated glycoside residues, including 1,2-linked-Rha*p* (3.06%), 1,3-linked-Ara*f* (5.30%), 1,5-linked-Ara*f* (3.38%), 1,3,5-linked-Ara*f* (5.47), 1,4-linked-Gal*p*A (63.78%), 1,4-linked-Glc (7.29%), T-Rha*p* (1.62%), T-Ara*f* (5.41%), and T-Gal*p*A (4.89%). The amount of Gal*p*A residues was calculated from the amount of increased galactosyl residues in the reduced polysaccharide compared with those in the native form. This result was consistent with the monosaccharide composition results. It is worth noting that Ara in PMPs-1 and PMPs-2 both existed as terminally linked arabinofuranosyl residues. 

### 3.4. FT-IR Spectrum Analysis of PMPs-1 and PMPs-2

**Figure 4:**
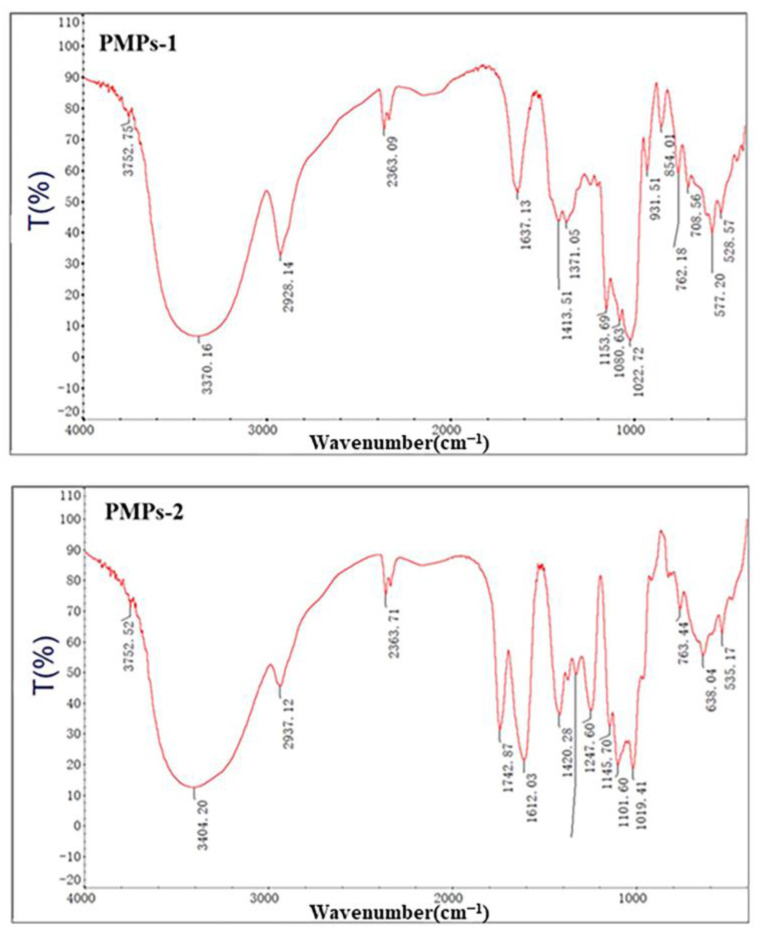
FT-IR spectra of PMPs-1 and PMPs-2.

The analysis of polysaccharide structures can be effectively accomplished through infrared absorption, which arises from molecular dipole moments or charge distribution induced by vibrational motions. This technique can yield valuable information regarding the structural properties of polysaccharides. Specifically, by examining characteristic absorption peaks in the infrared spectra of these compounds, the potential structural characteristics of individual components can be determined [24]. Figure 4 shows the infrared spectra of PMPs-1 and PMPs-2, providing a comprehensive visual representation of molecular vibrations and highlighting distinct features associated with their unique structural compositions.

The FT-IR spectrum of PMPs-1 revealed multiple signals spanning from 4000 to 400 cm^−1^, which are in line with the expected characteristics of polysaccharides. Notably, a strong and wide stretching peak at 3370 cm^−1^ was attributed to the vibrational motion of O-H groups, reflecting hydroxyl groups inherent to polysaccharides. Additionally, a relatively weaker stretching peak at 2928 cm^−1^ was caused by stretching vibrations of C-H bonds within the polysaccharide molecule [25]. Bands in the region around 1419 cm^−1^ corresponded to stretching vibrations due to carbon–oxygen (C-O) bonds [26]. Furthermore, a peak at 1022 cm^−1^ was attributed to angular vibrations involving oxygen–hydrogen (O-H) bonds. Another peak at 854 cm^−1^ is indicative of α-glycosidic linkages, which are important structural components [27]. Furthermore, an absorption peak at 1022 cm^−1^ suggests the presence of angular vibrations involving oxygen–hydrogen (O-H) bonds. Another absorption peak at 854 cm^−1^ is indicative of the presence of α-type glycosidic linkages, which are important structural components [28]. Lastly, a peak at 762 cm^−1^ may be associated with the symmetric ring stretching vibrations of the pyran ring, a characteristic feature of the molecular structure under investigation.

The FT-IR spectrum of PMPs-2 is depicted in Figure 4. The strong absorption peak of PMPs-2 at 3404 cm^−1^ was attributed to OH stretching vibrations due to hydrogen bonds of glycopyranose hydroxyl groups. Absorption peaks at 1742 cm^−1^ and 1612 cm^−1^ were attributed to the C=O stretching vibrations of the esterified carboxyl group (COO-R) and the asymmetric stretching vibrations of the carboxylic acid anion (COO-), respectively, suggesting uronic acid was included in the structure [29]. The peak at 1420 cm^−1^ may be caused by C-O stretching vibrations. The peak at 1329 cm^−1^ may result from the symmetric stretching vibrations of C=O. Finally, the peak at 1019 cm^−1^ may be attributed to O-H deformation vibrations.

### 3.5. SEM Analysis of PMPs-1 and PMPs-2

**Figure 5:**
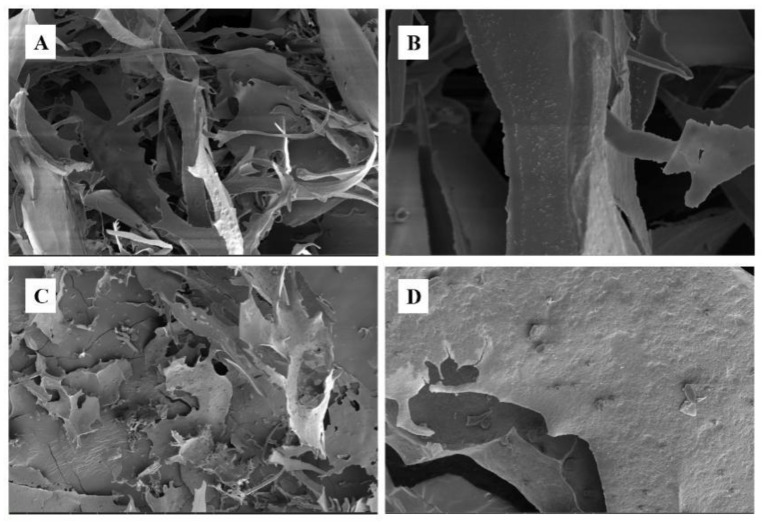
SEM images of PMPs-1 ((**A**) ×1000, (**B**) ×5000) and PMPs-2 ((**C**) ×1000, (**D**) ×5000).

The inherent complexity of polysaccharide structures gives rise to a multitude of microstructural variations. To gain insights into the surface morphology of polysaccharides, SEM has emerged as an investigational tool. Here, SEM was employed to assess the surface properties of PMPs-1 and PMPs-2 at different magnifications, including 1000- and 5000-fold, as depicted in Figure 5A–D. The SEM images captured for PMPs-1 and PMPs-2 offered visual information regarding their surface morphology. Notably, the images revealed that both samples predominantly consist of freely dispersed fragments rather than exhibiting a cohesive or organized structure. SEM images in Figure 5, specifically Figure 5A,B, depict a visual representation of PMPs-1. These images reveal that the surface of PMPs-1 exhibits both irregularity and smoothness, with a honeycomb-like structure. On the other hand, Figure 5C,D present SEM images for the acidic polysaccharide PMPs-2. Upon observation of these images, it is apparent that PMPs-2 is composed of layered structures with irregular shapes and relatively rough surfaces.

### 3.6. NMR Analysis of PMPs-1 and PMPs-2

**Figure 6:**
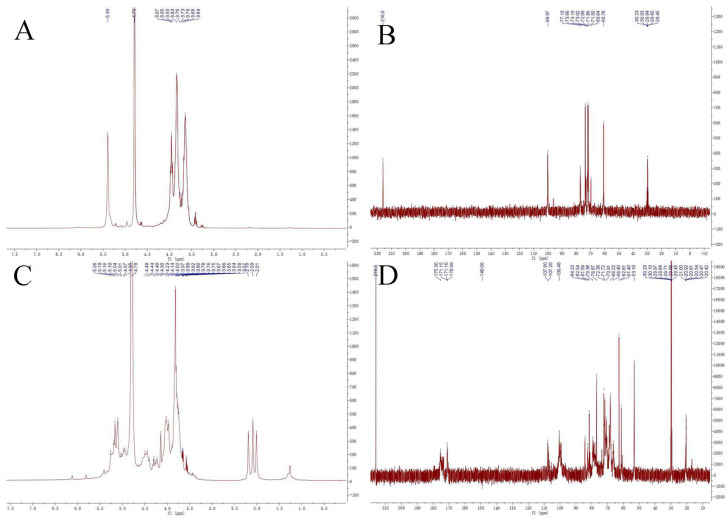
^1^H−NMR spectra (**A**,**C**) and ^13^C−NMR spectra (**B**,**D**) of the purified polysaccharides PMPs-1 and PMPs-2.

NMR analysis was employed to obtain more information about the structure of PMPs-1 and PMPs-2. Figure 6 shows the ^1^H and ^13^C NMR spectra of PMPs-1 and PMPs-2. Most of the proton signals were found at δ H 3.0~5.5 ppm and δ C 50~110 ppm, which were the typical NMR spectrums of polysaccharides. The number of anomeric proton signals at δ H 4.5~5.5 ppm and anomeric carbon signals at δ C 95~110 ppm reflects the types of monosaccharides. 

Signals at 5.60~4.90 and 4.90~4.30 ppm generally correspond to the anomeric protons of α- and β-anomers, respectively. The ^1^H NMR spectra of PMPs-1 in D_2_O are depicted in Figure 6A. Most of the proton signals appeared in the region of δ 3.1–5.4 ppm, and there were three peaks with strong intensity in the anomeric region (δ 4.5–5.9 ppm) with the chemical shifts of 5.30, 5.28, and 4.87 ppm, respectively. The results indicated that PMPs-1 contained α-glycosidic linkage, which is consistent with the results of FT-IR analysis and methylation GC-MS. The ^1^H NMR spectra of PMPs-2 in D_2_O are shown in Figure 6C. Anomeric signals observed at δ 4.88, 4.86 ppm and δ 5.72, 5.32, 5.17, 5.10, 5.07, and 5.01 ppm indicated that PMPs-2 contained α- and β-glycosidic linkages. Carbohydrate ring protons have signals mostly at 3.0~4.0 ppm because of the shielding effect of the OH group [30]. The ^13^C NMR spectra of PMPs-1 and PMPs-2 in D_6_O are shown in Figure 6B,D. Three signals appeared in the heterotopic carbon signal region of PMPs-1 (δ 99.97 ppm), versus eight signals in PMPs-2 (δ 107.80, 107.20, and 100.48 ppm). Four signals (δ 175.31, 171.15, 171.10, and 170.99 ppm) appeared in the ^13^C NMR spectrum of PMPs-2, which are characteristic of the carboxyl group of uronic acid. Carbon signals between δ 60.73 and δ 77.10 ppm reflect absorption signals for C_2_–C_6_ in monosaccharides in PMPs-1, versus signals between δ 53.19 and δ 84.22 ppm reflect absorption signals for C_2_–C_6_ in PMPs-1. The NMR analysis corroborates monosaccharide composition and infrared analysis.

### 3.7. Immunomodulatory Activities of PMPs-1 and PMPs-2

#### 3.7.1. Effects of PMPs-1 and PMPs-2 on Cell Viability

**Figure 7:**
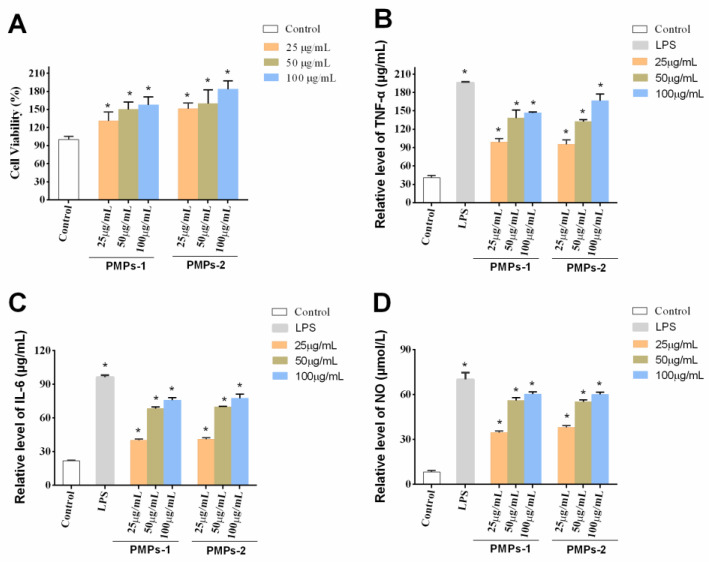
Effects of PMPs-1 and PMPs-2 on the viability (**A**), production of TNF-α (**B**), production of IL-6 (**C**), and production of NO (**D**) in RAW264.7 cells. * *p* < 0.05 denotes a statistically significant difference between the treated and control groups.

As cells trigger innate immunity against infections and inflammation, macrophages constitute a great cellular model for assessing the body’s capacity to regulate the immune system. Their significance lies in their ability to serve as key players in immune responses, enabling a deeper understanding of intricate mechanisms underlying immune regulation within the body [31]. Polysaccharides typically exert their immune regulatory activities by affecting macrophages. RAW 264.7 macrophages, the prevailing model for investigating immunomodulatory functions, have been extensively utilized in previous research. Notably, polysaccharides from Atractylodis Macrocephalae Rhizoma [32], Radix Adenophorae [33], and Rehmanniae Radix Praeparata [34] exhibit potent immunoregulatory activity by promoting cell proliferation of RAW 264.7 cells. The current research aimed to investigate the impacts of PMPs-1 and PMPs-2 on cell proliferation of RAW 264.7. Remarkably, versus the blank control group, PMPs-1 and PMPs-2 exerted negligible toxic effects. Moreover, at 25–100 μg/mL (*p* < 0.05), both PMPs-1 and PMPs-2 enhanced macrophage proliferation. These findings, visually represented in Figure 7A, underscore the stimulatory effects of PMPs-1 and PMPs-2 on macrophages.

#### 3.7.2. Effects of PMPs-1 and PMPs-2 on the Contents of Cytokines in RAW264.7 Cells

Cytokines, encompassing diverse bioactive proteins, are synthesized through the collaborative effort of immune and non-immune cells [35]. Among these cytokines, notable examples include NO, IL-6, and TNF-α [36]. Functioning as messenger molecules and cytokines play pivotal roles in multiple pathophysiological events associated with the immune response [37,38]. NO assumes a central position as a key mediator within the intricate network of macrophages. Its involvement spans essential immune-related functions and is indispensable for the immune system’s ability to combat invading pathogens [39]. In the context of tissue damage from bacterial, fungal, or tumor cell assaults, macrophages exhibit a remarkable defense mechanism involving the release of large amounts of NO [40]. This immune response constitutes an important line of defense against pathogenic organisms. Intriguingly, in Figure 7B, it is evident that NO levels displayed a significant and statistically discernible difference at concentrations of 25–100 μg/mL for PMPs-1 and PMPs-2 versus control values (*p* < 0.05). TNF-α, considered a pleiotropic cytokine, plays a pivotal role in orchestrating inflammation and eliciting immune responses [41]. Within the complex framework of immune processes, IL-6 plays a vital role. This multifunctional cytokine is highly involved in various immunological activities, including phagocytosis, antigen presentation, and the fine-tuning of inflammatory responses [42]. A plethora of studies have conclusively exhibited the ability of polysaccharides to exert regulatory effects on cytokine and chemokine production. For example, a novel acidic polysaccharide named SSPA5-1 was isolated from *Scapharca subcrenata* in Li’s study. The immunoregulatory activity of SSPA50-1 was evaluated on RAW 264.7 cells, and the results showed that SSPA50-1 possessed potent immunoregulatory activity by enhancing the NO, iNOS, TNF-T, and IL-6 secretion capacity of RAW 264.7 cells [43]. Sun et al. studied the immunomodulatory activity of polysaccharides from *Helicteres angustifolia* L. on RAW 264.7 cells. They found that SPF3-1 can stimulate the NO and immunomodulatory cytokines generation of RAW 264.7 cells, such as TNF-α, IL-2, IL-4, IL-6, and IL-10 [44]. Notably, these polysaccharides stimulate the release of a wide spectrum of cytokines, encompassing both anti-inflammatory and pro-inflammatory groups. Consequently, these dynamic molecular entities play essential roles in orchestrating the delicate balance of inflammatory responses within the complex network of immune processes [45,46]. The results are shown in Figure 7C,D. Notably, RAW 264.7 cells administered PMPs-1 and PMPs-2 at 25 to 100 μg/mL, which showed remarkably upregulated TNF-α and IL-6 (*p* < 0.01).

The complex associations of the immune activities of polysaccharides and their structural properties are widely recognized in the scientific community, encompassing factors such as molecular size, sulfation levels, carboxyl groups, and molecular conformation. These structural characteristics control the immunomodulatory effects of polysaccharides [47,48]. For instance, when examining the pro-inflammatory response elicited in macrophages by polysaccharides from Cystoseira indica, a close association was found between low *M*w, sulfation content, and activity [49]. Cui et al. isolated and purified three water-soluble polysaccharides (AMAP-1, AMAP-2, and AMAP-3) from Atractylodis Macrocephalae Rhizoma. The primary immunomodulatory activity of three polysaccharides in RAW 264.7 macrophages was investigated. The results showed that AMAP-1 and AMAP-2 with high Mw can stimulate RAW 264.7 macrophages to release NO, but low *M*w AMAP-3 rich in homogalacturonan cannot [32]. In this study, the regulatory effects of PMPs-1 and PMPs-2 on immune function were examined. The findings revealed that both PMPs-1 and PMPs-2 exerted positive effects on the immune system by stimulating TNF-α, IL-6, and NO secretion in RAW 264.7 cells. Notably, these stimulatory effects were dose-dependent at 25 to 100 μg/mL. Importantly, PMPs-1 and PMPs-2 had no significant differences in their regulatory abilities in this investigation of RAW 264.7 cells. When studying the immunomodulatory effects of plant polysaccharides with different structures using in vitro cell models, there may not be significant differences in their activity. In Zhou’s study, two polysaccharides (SDH-WA and SDH-0.2A) from Rehmanniae Radix Praeparata were extracted and purified. The immunomodulatory effects of two polysaccharides were investigated by using RAW 264.7 cells. The results showed that both polysaccharides showed significantly promoted phagocytic activity, but there were no obvious differences between neutral polysaccharide SDH-WA and acidic polysaccharide SDH-0.2A. This research result is similar to our research findings. However, in vitro data do not always translate into in vivo findings [34]. In Zhang’s study [13], two purified polysaccharides from PM, namely, WPMP-1 and WPMP-2, exhibited activating effects on splenocytes and macrophages. However, the acid polysaccharide WPMP-2 exhibited better immunomodulatory activity than the neutral polysaccharide WPMP-1, which indicates that the positive effects of polysaccharides may be associated with a higher content of uronic acid. Therefore, further investigation is required in the future to unveil the associated mechanism and structure–activity relationship for these polysaccharides.

## 4. Conclusions

In summary, two polysaccharides (PMPs-1 and PMPs-2) were obtained from the traditional Chinese medicine herb *Polygonum multiflorum* Thunb. The structural characterization was determined by different methods, such as UV, HPLC-PDA, SEM, FT-IR, GC-MS, HPGPC, and NMR. These two polysaccharides possessed different monosaccharide compositions and *M*w; PMPs-1 is a neutral heteropolysaccharide, which is composed of Man, Glc, Gal, and Ara at molar ratios of 0.9, 78.6, 1.0, and 1.6, and with *M*w of 255.5 kDa. The PMPs-2 is an acidic heteropolysaccharide, which is composed of Rha, GalA, Glc, Gal, and Ara with molar ratios of 1.6, 20.3, 2.7, 1.0, and 6.3, and with *M*w of 55.7 kDa. In previous studies on the activity of PM polysaccharides, the focus was mainly on antioxidant activity analysis, with less research involving immunomodulatory effects. In the existing analysis of the immune activity of PM polysaccharides, in vitro and in vivo immunosuppressive models are mainly used. For example, Zhang et al. used a 5-Fu-induced macrophage immunosuppressive model and Chen et al. [50] used a cyclophosphamide-induced mice immunosuppressive model to reveal the immunomodulatory effects of PM polysaccharides. In our study, the RAW 264.7 macrophage model, a widely used model for studying the immune activity of polysaccharides, was used for studying the immunomodulatory effects of PM. The immunomodulatory activity assay showed that PMPs-1 and PMPs-2 (25~100 μg/mL) markedly enhance cell proliferation in RAW 264.7 cells. In addition, PMPs-1 and PMPs-2 increase the phagocytic activity of macrophages and starkly promote the secretion of NO and cytokines (TNF-α and IL-6) dose-dependently. In summary, these two polysaccharides can be explored as a new food additive and immune enhancer. These findings also provide a basis for further application of PM in food and pharmaceutical industries.

## Figures and Tables

**Table 1:** Methylation analysis of PMPs-1 and PMPs-2.

Sample	Retention Time (min)	Linkage Pattern	Mass Fragments (*m*/*z*)	Peak Area Ratio (%)
PMPs-1	13.840	T-Glc*p*	87, 101, 102, 118, 129, 145, 161, 162, 205	8.03
	18.014	1,6-Linked-Glc*p*	102, 113, 118, 129, 173, 233	2.81
	18.295	1,4-Linked-Glc*p*	87, 99, 102, 113, 118, 129, 173, 233	80.54
	21.707	1,4,6-Linked-Glc*p*	118, 261	8.62
PMPs-2	10.560	T-Rha*p*	89, 102, 118, 131, 162, 175	1.62
	10.950	T-Ara*f*	87, 102, 118, 129, 145, 161, 162	5.41
	13.485	1,2-Linked-Rha*p*	89, 100, 115, 130, 131, 190	3.06
	13.830	1,3-Linked-Ara*f*	87, 101, 102, 118, 129, 145, 161, 162, 205	5.30
	14.680	T-Gal*p*A	87, 102, 118, 129, 161, 162, 205	4.89
	15.125	1,5-Linked-Ara*f*	87, 102, 118, 129, 189	3.38
	17.752	1,3,5-Linked-Ara*f*	85, 99, 118, 127	5.47
	17.785	1,4-Linked-Gal*p*(A)	87, 99, 102, 113, 118, 131, 175	63.78
	18.285	1,4-Linked-Glc*p*	87, 99, 102, 113, 118, 129, 233	7.29

## Data Availability

The original contributions presented in the study are included in the article/Supplementary Material, further inquiries can be directed to the corresponding authors.

## Supplementary Materials

The following supporting information can be downloaded at https://www.mdpi.com/article/10.3390/foods13060932/s1, Supplementary File S1: The mass spectrums of methylation analysis of each linkage.
