# Supplementary material for: Purification and Structural Characterization of Polysaccharides from Polygonum multiflorum Thunb. and Their Immunostimulatory Activity in RAW264.7 Cells
Source: Foods. 2024 Mar 19;13(6):932. doi: 10.3390/foods13060932 (PMC10969798; doi:10.3390/foods13060932)
Supplement: Supplementary file 1 [file foods-13-00932-s001.zip › foods-2803435-supplementary.pdf]

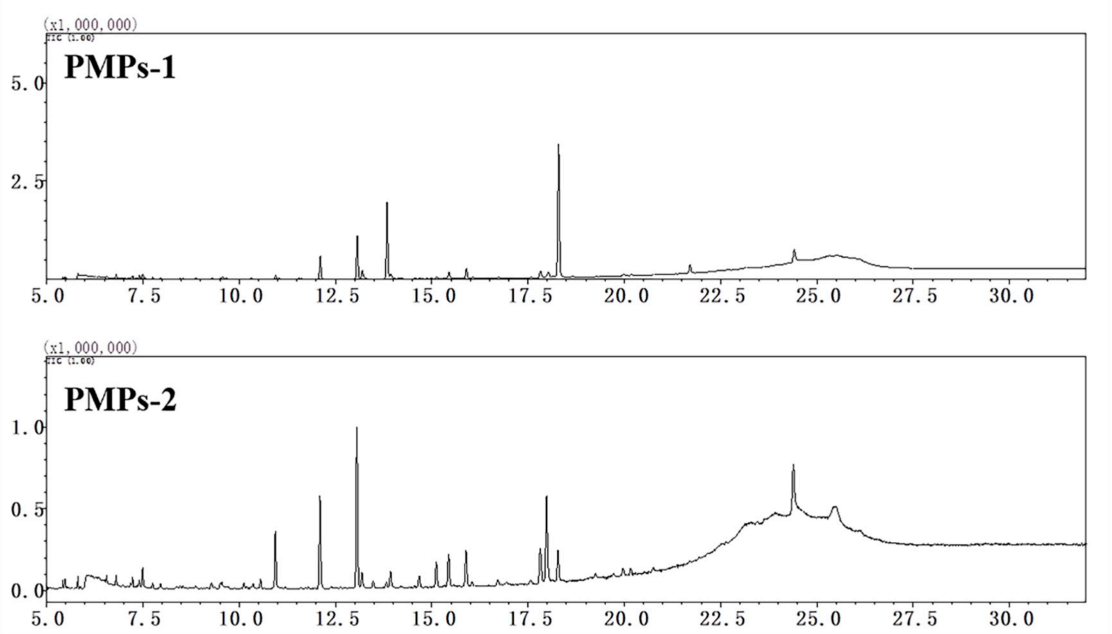

Figure S1. The GC-MS total ion chromatograms of PMPs-1 and PMPs-2.

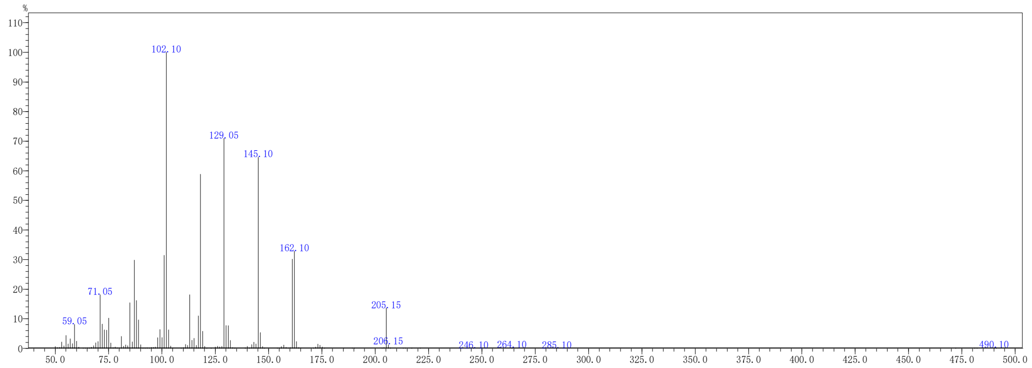

Figure S2. Mass spectrum of methylated fragments of T-Glc*p*.

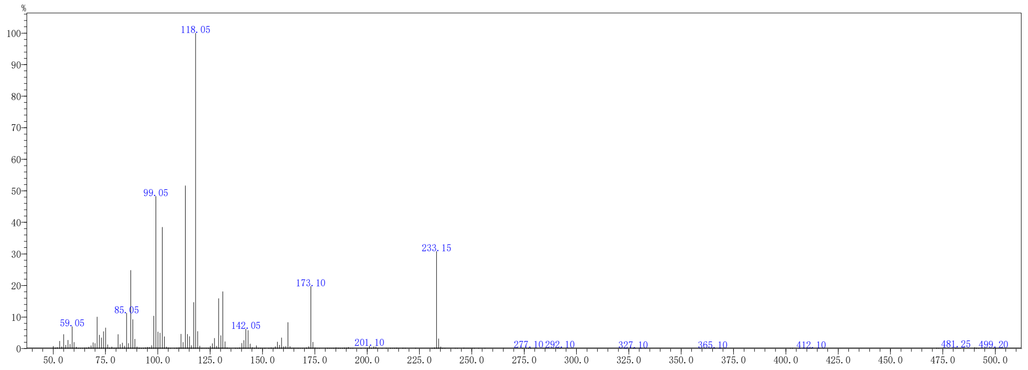

Figure S3. Mass spectrum of methylated fragments of 1,4-linked-Glc*p*.

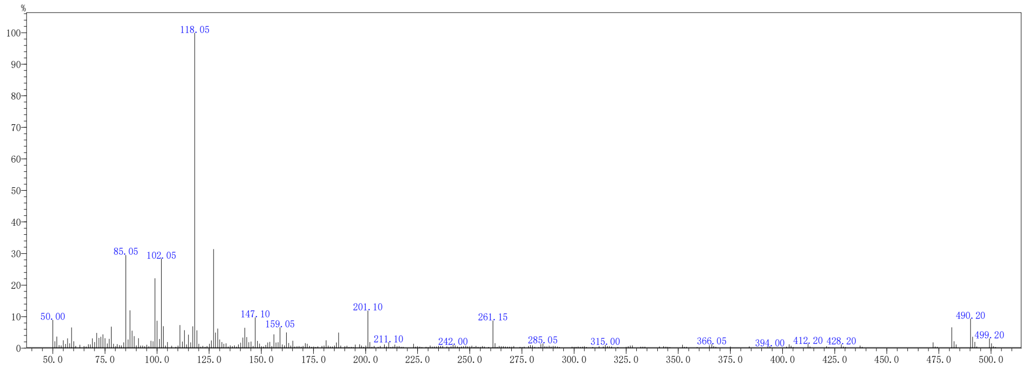

Figure S4. Mass spectrum of methylated fragments of 1,3-linked-Ara*f*.

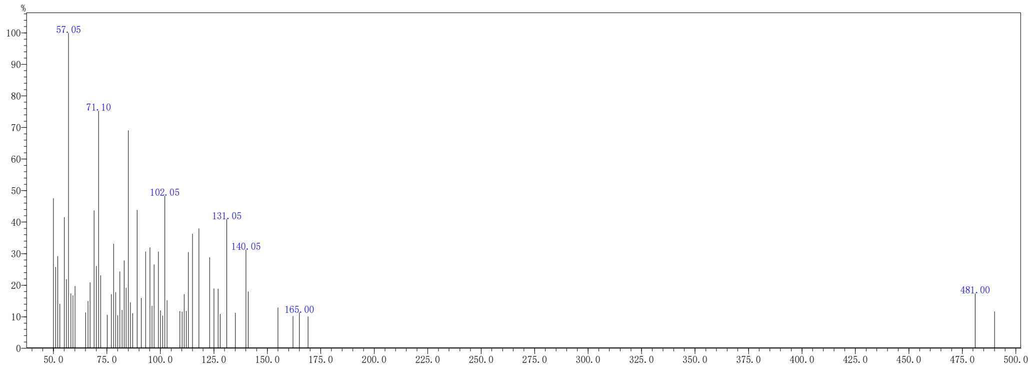

Figure S5. Mass spectrum of methylated fragments of T-Rha*p*.

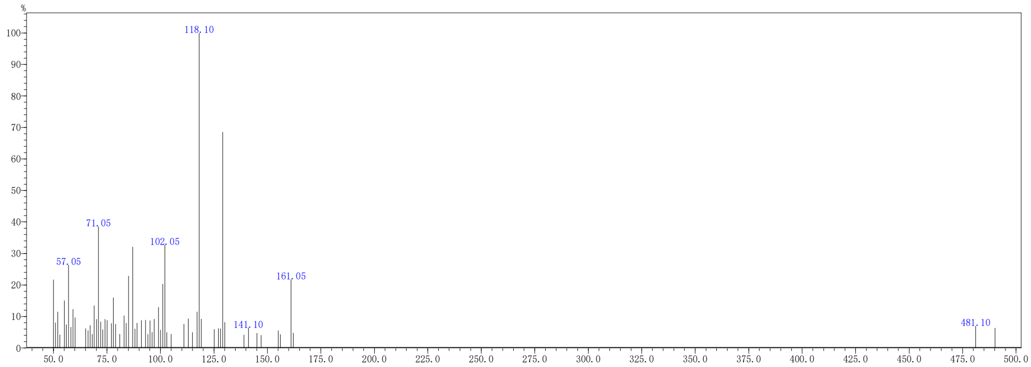

Figure S6. Mass spectrum of methylated fragments of T-Ara*f*.

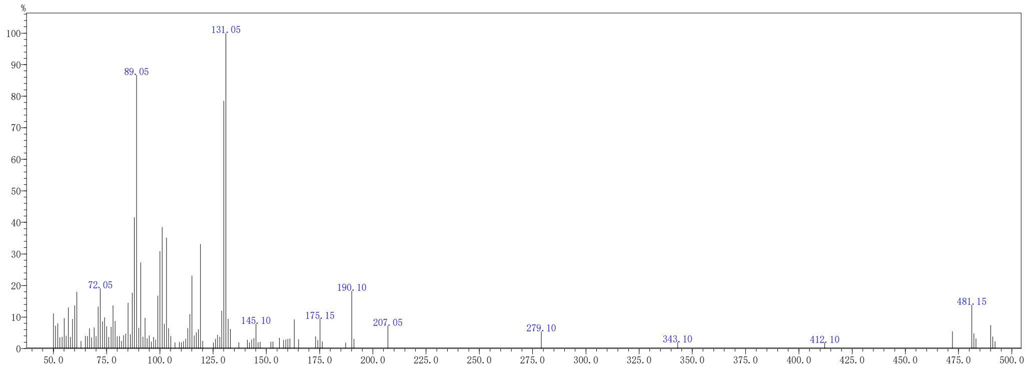

Figure S7. Mass spectrum of methylated fragments of 1,2-linked-Rha*p*.

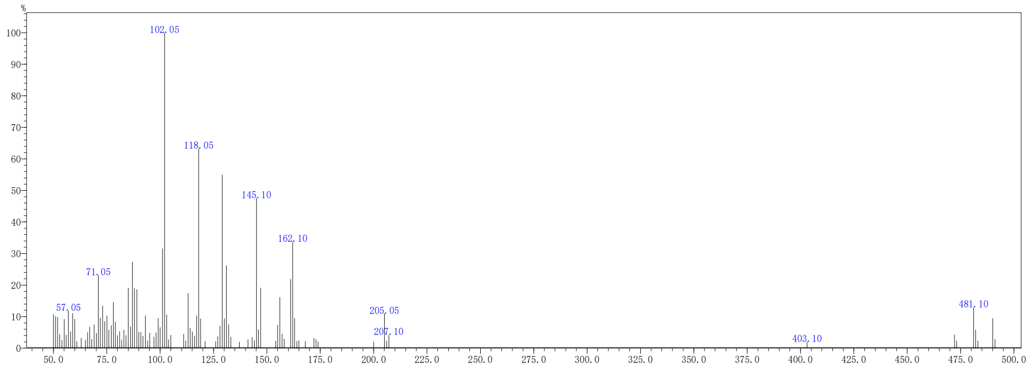

Figure S8. Mass spectrum of methylated fragments of T-Gal*p*.

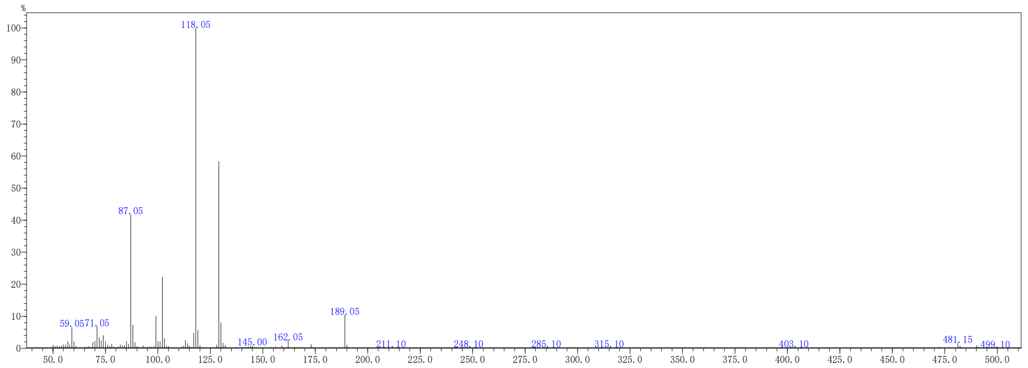

Figure S9. Mass spectrum of methylated fragments of 1,5-linked-Ara*f*.

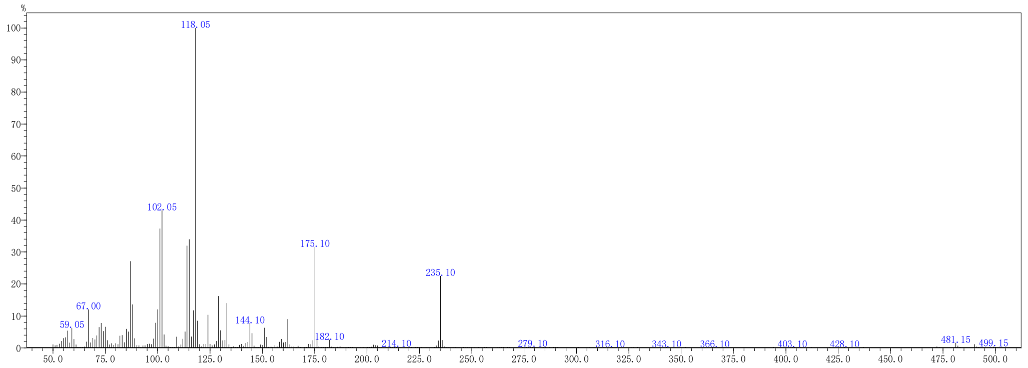

Figure S10. Mass spectrum of methylated fragments of 1,3,5-linked-Ara*f*.

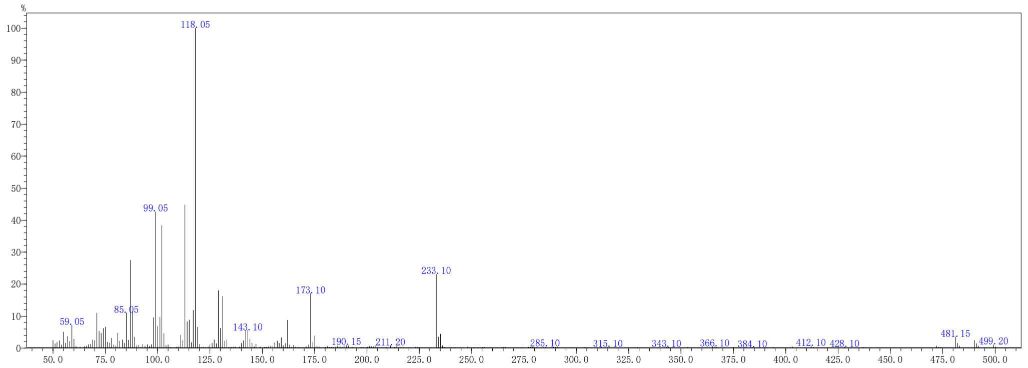

Figure S11. Mass spectrum of methylated fragments of 1,4-linked-Gal*p*.
